# Body composition and checkpoint inhibitor treatment outcomes in advanced melanoma: a multicenter cohort study

**DOI:** 10.1093/jnci/djaf039

**Published:** 2025-02-20

**Authors:** Mark Schuiveling, Laurens S Ter Maat, Isabella A J Van Duin, Rik J Verheijden, Max F Troenokarso, Pim Moeskops, Joost J C Verhoeff, Sjoerd G Elias, Wouter A C van Amsterdam, Femke Burgers, Franchette W P J Van den Berkmortel, Marye J Boers-Sonderen, Martijn F Boomsma, Jan Willem De Groot, John B A G Haanen, Geke A P Hospers, Djura Piersma, Gerard Vreugdenhil, Hans M Westgeest, Ellen Kapiteijn, Mariette Labots, Wouter B Veldhuis, Paul J Van Diest, Pim A De Jong, Josien P W Pluim, Tim Leiner, Mitko Veta, Karijn P M Suijkerbuijk

**Affiliations:** Department of Medical Oncology, University Medical Center Utrecht, Utrecht University, Utrecht, The Netherlands; Image Sciences Institute, University Medical Center Utrecht, Utrecht University, Utrecht, The Netherlands; Department of Medical Oncology, University Medical Center Utrecht, Utrecht University, Utrecht, The Netherlands; Department of Medical Oncology, University Medical Center Utrecht, Utrecht University, Utrecht, The Netherlands; Department of Epidemiology, Julius Center for Health Sciences and Primary Care, University Medical Center Utrecht, Utrecht University, Utrecht, The Netherlands; Department of Medical Oncology, University Medical Center Utrecht, Utrecht University, Utrecht, The Netherlands; Quantib (currently DeepHealth), Rotterdam, The Netherlands; Department of Radiotherapy, University Medical Center Utrecht, Utrecht University, Utrecht, The Netherlands; Department of Epidemiology, Julius Center for Health Sciences and Primary Care, University Medical Center Utrecht, Utrecht University, Utrecht, The Netherlands; Department of Data Science and Biostatistics, Julius Center for Health Sciences and Primary Care, University Medical Center Utrecht, Utrecht University, Utrecht, The Netherlands; Department of Medical Oncology, Netherlands Cancer Institute, Amsterdam, The Netherlands; Department of Medical Oncology, Zuyderland Medical Center, Sittard-Geleen, The Netherlands; Department of Medical Oncology, Radboudumc, Radboud University, Nijmegen, The Netherlands; Department of Radiology, Isala Zwolle, Zwolle, The Netherlands; Isala Oncology Center, Isala Zwolle, Zwolle, The Netherlands; Department of Medical Oncology, Netherlands Cancer Institute, Amsterdam, The Netherlands; Department of Medical Oncology, Leiden University Medical Center, Leiden University, Leiden, The Netherlands; Melanoma Clinic, Centre Hospitalier Universitaire Vaudois, Lausanne, Switzerland; Department of Medical Oncology, UMC Groningen, University of Groningen, Groningen, The Netherlands; Department of Medical Oncology, Medisch Spectrum Twente, Enschede, The Netherlands; Department of Medical Oncology, Maxima Medical Center, Veldhoven, The Netherlands; Department of Internal Medicine, Amphia Hospital, Breda, The Netherlands; Department of Medical Oncology, Leiden University Medical Center, Leiden University, Leiden, The Netherlands; Department of Medical Oncology, UMC, Vrije Universiteit Amsterdam, Cancer Center Amsterdam, Amsterdam, The Netherlands; Quantib (currently DeepHealth), Rotterdam, The Netherlands; Department of Radiology, University Medical Center Utrecht, Utrecht University, Utrecht, The Netherlands; Department of Pathology, University Medical Center Utrecht, Utrecht University, Utrecht, The Netherlands; Department of Radiology, University Medical Center Utrecht, Utrecht University, Utrecht, The Netherlands; Image Sciences Institute, University Medical Center Utrecht, Utrecht University, Utrecht, The Netherlands; Medical Image Analysis, Department of Biomedical Engineering, Eindhoven University of Technology, Eindhoven, The Netherlands; Quantib (currently DeepHealth), Rotterdam, The Netherlands; Department of Radiology, Mayo Clinical, Rochester, MN, United States; Medical Image Analysis, Department of Biomedical Engineering, Eindhoven University of Technology, Eindhoven, The Netherlands; Department of Medical Oncology, University Medical Center Utrecht, Utrecht University, Utrecht, The Netherlands

## Abstract

**Background:**

The association of body composition with checkpoint inhibitor outcomes in melanoma is a matter of ongoing debate. In this study, we aim to investigate body mass index (BMI) alongside computed tomography (CT)-derived body composition metrics in the largest cohort to date.

**Methods:**

Patients treated with first-line anti-PD1 ± anti-CTLA4 for advanced melanoma were retrospectively identified from 11 melanoma centers in The Netherlands. From baseline CT scans, 5 body composition metrics were extracted: subcutaneous adipose tissue index, visceral adipose tissue index, skeletal muscle index, density, and gauge. These metrics were correlated in univariable and multivariable Cox proportional hazards analysis with progression-free survival, overall survival, and melanoma-specific survival (PFS, OS, and MSS).

**Results:**

A total of 1471 eligible patients were included. Median PFS and OS were 9.1 and 38.1 months, respectively. Worse PFS was observed in underweight patients (multivariable hazard ratio [HR] = 1.86, 95% CI = 1.14 to 3.06). Furthermore, prolonged OS was observed in patients with higher skeletal muscle density (multivariable HR = 0.88, 95% CI = 0.81 to 0.97) and gauge (multivariable HR = 0.61, 95% CI = 0.82 to 0.998), whereas higher visceral adipose tissue index was associated with worse OS (multivariable HR = 1.12, 95% CI = 1.04 to 1.22). No association with survival outcomes was found for overweight, obesity, or subcutaneous adipose tissue.

**Conclusion:**

Our findings suggest that underweight BMI is associated with worse PFS, whereas higher skeletal muscle density and lower visceral adipose tissue index were associated with improved OS. These associations were independent of known prognostic factors, including sex, age, performance status, and extent of disease. No significant association between higher BMI and survival outcomes was observed.

## Introduction

The introduction of immune checkpoint inhibitors (ICI) has revolutionized advanced melanoma care. The prognosis for advanced melanoma was historically very poor, with a 1-year overall survival (OS) of less than 25%.^1^ In contrast, patients treated in the CheckMate 067 trial with antiprogrammed cell death 1 (anti-PD1) had a 6.5-year OS rate of 43%. Patients treated with both anti-PD1 and anticytotoxic T-lymphocyte associated protein-4 (anti-CTLA4) antibodies even had a 6.5-year OS rate of 57%.^2^

However, many open questions remain about how checkpoint inhibitors interact with tumor and host. Both anti-CTLA4 and anti-PD1 antibodies block proteins that inhibit the immune response, which leads to increased immune activity against the tumor.^3^ Although some mechanisms of primary resistance have been identified,^4^ it is not fully understood why some patients progress during treatment, whereas others do not.

One such open question is the association between obesity and checkpoint inhibitor treatment outcomes. On the one hand, several pan-cancer meta-analyses published in 2020 and 2021 reported better survival outcomes in patients with obesity compared to patients with normal body mass index (BMI).^5-7^ This association, dubbed the “obesity paradox,” was also found to be significant in the subgroup of studies on patients with melanoma.^6^^,^^7^ On the other hand, an updated meta-analysis by Roccuzzo et al. in melanoma concluded that the prognostic value of BMI could not be confirmed due to the limited available evidence.^8^ This indicates that the topic of obesity and checkpoint inhibitor treatment outcomes is an area of ongoing research where more high-quality evidence is needed.

In addition to BMI, previous works investigated computed tomography (CT)-derived body composition metrics. These metrics include the amount and density of skeletal muscle and the amount of subcutaneous and adipose tissue.^9^ Due to advances in deep learning for automatic image analysis, this category of predictors has become increasingly prominent in research in recent years.^10^^,^^11^ The advantage of these metrics is that they can more accurately capture a patient’s body composition, making it possible to assess body composition states such as sarcopenia or a high amount of visceral fat, whereas with BMI, weight may misrepresent patients with high muscle mass. In addition, with BMI it is not possible to distinguish patients with high visceral or subcutaneous adipose tissue. Previous studies on body composition metrics, however, reported differing results and have some methodological limitations, most notably a limited sample size.^12^

Several mechanisms have been proposed for explaining associations between body composition and checkpoint inhibitor outcomes. Lower skeletal muscle density could be the result of cancer-related cachexia, a condition associated with diminished OS.^9^ In contrast, increased PD1 expression was noted in obese patients with melanoma.^10^

Due to inconsistent results from previous research on BMI and CT-derived body composition metrics in advanced melanoma patients treated with ICI, their association with survival remains uncertain. Therefore, this study aimed to investigate these associations in the largest cohort of melanoma patients treated with ICI to date.

## Methods

### Patient selection

Patients were eligible if they were (1) more than 18 years of age, (2) treated for unresectable stage IIIC or stage IV cutaneous melanoma with (3) first-line anti-PD1 with or without CTLA4 inhibition (4) between January 1, 2016, and February 1, 2023. Disease stage was defined as based upon the AJCC 8th edition.^1^ Patients were excluded if (1) no baseline CT scan was available, (2) no transverse slice of the third lumbar vertebrae was in the field of view of the scan, (3) metal artifacts were present at the L3 level or (4) patient height or weight at baseline was unavailable. Eligible patients from 11 melanoma treatment centers in The Netherlands (Amphia Breda, Amsterdam UMC, Isala Zwolle, Leiden University Medical Center, Maxima MC, Medisch Spectrum Twente, Netherlands Cancer Institute, Radboudumc, University Medical Center Groningen, University Medical Center Utrecht, Zuyderland) were identified using high-quality registry data. This study was deemed not subject to the Medical Research Involving Human Subjects Act according to Dutch regulations by the Medical Ethics Committee; informed consent was waived.

### BMI and clinical predictors

**Figure 1.**
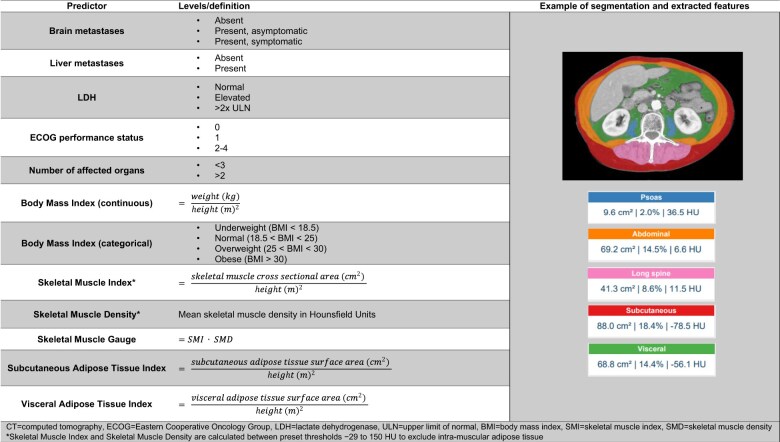
Definition of the included predictors and evaluated models.

Height and weight at baseline were extracted from electronic patient files and were used to calculate BMI. In addition, several previously identified clinical predictors of checkpoint inhibitor treatment outcomes in advanced melanoma were extracted. These were (1) Eastern Cooperative Oncology Group (ECOG) performance status, (2) level of lactate dehydrogenase (LDH), presence of (3) brain and (4) liver metastases, and (5) number of affected organs^13-15^ (categories are shown in Figure 1).

### CT body composition metrics extraction

Metrics were obtained using Quantib Body Composition version 0.2.1, a dedicated deep learning segmentation algorithm that has proven to achieve high correspondence to manual segmentations in previous studies.^16-18^ First, all baseline CT scans were resampled to a slice thickness of 5 mm. Subsequently, the slice in the middle of the third lumbar vertebra^19^ was automatically selected using a convolutional neural network. On the 5 consecutive slices centered around this selected slice, the following compartments were automatically segmented using a second convolutional neural network: psoas, abdominal and long spine muscles (together making up the skeletal muscles), subcutaneous adipose tissue, and visceral adipose tissue. All segmentations were manually reviewed and corrected where necessary (performed by the author L.S.T.M. while being blinded to the outcome). Based on these segmentations, 5 commonly used metrics^9^^,^^20-22^ were calculated using the definitions in Figure 1: skeletal muscle index (SMI), skeletal muscle density (SMD), skeletal muscle gauge (SMG), subcutaneous adipose tissue index (SATI), and visceral adipose tissue index (VATI). All metrics were normalized to zero mean and unit SD to facilitate interpretation. Since SMD and SMG differed significantly between patients who underwent a contrast-enhanced CT scan vs those who underwent a noncontrast CT scan, SMD and SMG were normalized separately for both groups.

Skeletal muscle density and SMI were used to classify patients as having myosteatosis or sarcopenia. Classification is based on the cutoffs established by Martin et al.^22^ Myosteatosis was defined based on SMD, with thresholds of <41 Hounsfield Units (HU) for both men and women with a BMI <25, and <33 HU for both men and women with a BMI ≥25. Sarcopenia was defined based on SMI thresholds: for men, SMI <43 cm^2^/m^2^ if BMI <25 and <53 cm^2^/m^2^ if BMI ≥25; for women, SMI <41 cm^2^/m^2^ regardless of BMI.

### Outcome definition

The primary endpoints were progression-free survival (PFS) and OS. Progression-free survival was defined as the time from the start of treatment to progression or death; OS was defined as time from the start of treatment to death due to any cause. The secondary outcome was melanoma-specific survival (MSS), defined as the time from the start of treatment to death from melanoma. Patients not reaching the endpoint were right censored at the date of the last contact, or when a different treatment was initiated.

### Statistical analysis

Correlation among body composition variables was assessed using Pearson’s correlation coefficient. The association between body composition metrics and outcomes was assessed using univariable and multivariable Cox proportional hazard models. In multivariable analyses, a separate model was constructed for every body composition metric, combined with previously identified clinical factors (ECOG performance status, level of LDH, presence of brain and liver metastases, and number of affected organs). Body mass index was assessed as a categorical variable, using the established cutoffs for underweight (<18.5), normal (between 18.5 and 25), overweight (between 25 and 30), and obese (>30). Multiple imputation was performed using the MICE R package with 21 imputations. Subgroup analyses were conducted for patients treated with monotherapy (anti-PD1) and combination therapy (anti-PD1 + anti-CTLA4). Unless stated otherwise, 95% confidence intervals (CIs) are displayed.

## Results

### Patient characteristics

Out of 1944 eligible patients, 1471 patients (76%) were included (Figure S1). Characteristics of the included patients are shown in Table 1; these characteristics were similar to those of excluded patients (Table S1). Median PFS and OS were 9.1 and 38.1 months, respectively. Median MSS was not reached. The subgroups of patients treated with anti-PD1 monotherapy and anti-PD1 plus anti-CTLA-4 combination therapy consisted of 942 (64%) and 529 (36%) patients, respectively (Table S2). Subgroups of patients who underwent noncontrast CT (in combination with 18-fluorodeoxyglucose positron emission tomography) vs contrast-enhanced consisted of 611 and 860 patients, respectively.

**Table 1.** Characteristics of the included patients.

Characteristic	Total, No. *n* = 1471
Age, mean (SD)	65.1 (13.0)
Sex, *n* (%)	
Female	579 (39.4)
Male	892 (60.6)
Therapy, *n* (%)	
Anti-PD1	942 (64.0)
Ipilimumab and nivolumab	529 (36.0)
Scan type, *n* (%)	
Contrast-enhanced	860 (58.5)
No contrast	611 (41.5)
Stage, *n* (%)	
IIIC	131 (8.9)
IV M1a	130 (8.8)
IV M1b	217 (14.8)
IV M1c	639 (43.4)
IV M1d	344 (23.4)
Missing	10 (0.7)
ECOG performance status, *n* (%)	
0	798 (54.2)
1	489 (33.2)
2-4	110 (7.5)
Missing	74 (5.0)
Brain metastases, *n* (%)	
Absent	952 (64.7)
Asymptomatic	212 (14.4)
Symptomatic	132 (9.0)
Missing	175 (11.9)
Liver metastases, *n* (%)	
Absent	939 (63.8)
Present	379 (25.8)
Missing	153 (10.4)
LDH, *n* (%)	
Normal	1013 (68.9)
1-2× ULN	330 (22.4)
>2× ULN	110 (7.5)
Missing	18 (1.2)
Number of affected organs, *n* (%)	
<3	886 (60.2)
>2	585 (39.8)
Body Mass Index, *n* (%)	
Underweight	21 (1.4)
Normal	604 (41.1)
Overweight	586 (39.8)
Obese	260 (17.7)
Skeletal muscle index, median [Q1, Q3]	44.4 [38.7,50]
Skeletal muscle density, median [Q1, Q3]	30.9 [23.4,37.8]
Skeletal muscle gauge, median [Q1, Q3]	1370.5 [984.4,1782.9]
Subcutaneous adipose tissue index, median [Q1, Q3]	51.7 [37.6,72.9]
Visceral adipose tissue index, median [Q1, Q3]	47.6 [26.6,73.3]
Median overall survival (months)	38.1
Median progression-free survival (months)	9.1
Median melanoma-specific survival (months)	Not reached

Abbreviations: ECOG = Eastern Cooperative Oncology Group, LDH = lactate dehydrogenase, ULN = upper limit of normal.

### Body mass index

**Figure 2.**
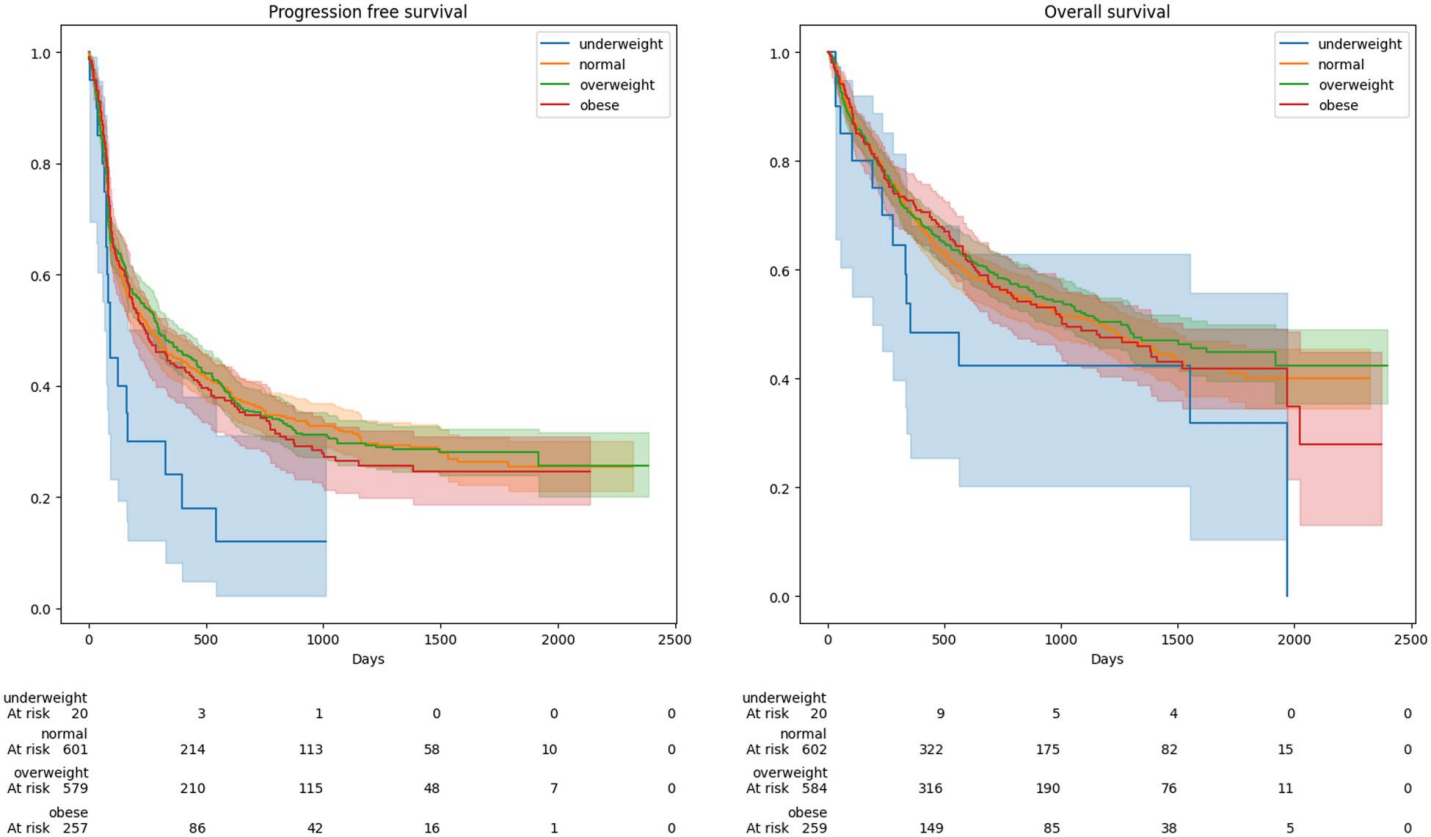
Kaplan-Meier curves for progression-free and overall survival in patients treated with immune checkpoint inhibition for advanced melanoma according to BMI subgroup. Abbreviation: BMI = body mass index.

Out of 1471 patients, 21 (1.4%) were underweight, 604 (41.1%) had normal BMI, 586 (39.8%) were overweight, and 260 (17.7%) were obese. Underweight patients had significantly worse PFS than patients with normal weight in both univariable and multivariable analyses (multivariable HR = 1.87, 95% CI = 1.14 to 3.07, Table 2, Figure 2). A similar, but statistically nonsignificant association was observed for OS (multivariable HR = 1.57, 95% CI = 0.89 to 2.77, Table 3, Figure 2). Underweight patients had more advanced disease, worse ECOG performance status, higher levels of LDH at baseline, a higher prevalence of liver and (symptomatic) brain metastasis and were less likely to receive combination therapy (Table S3). Overall survival and PFS were not significantly different in overweight or obese patients when compared to normal BMI patients (Tables 2-4).

**Table 2.** Univariable and multivariable Cox proportional hazard models for progression-free survival.

	Univariable		Multivariable^a^	
	HR^b^ (95% CI)	*P*	HR^b^ (95% CI)	*P*
Body mass index				
Underweight	1.83 (1.12 to 2.97)	.015	1.86 (1.13 to 3.06)	.014
Normal	1.00		1.00	
Overweight	0.99 (0.86 to 1.13)	.853	1.00 (0.87 to 1.15)	.971
Obese	1.03 (0.87 to 1.24)	.702	1.11 (0.93 to 1.33)	.247
Skeletal muscle index	1.03 (0.96 to 1.09)	.432	1.08 (0.99 to 1.16)	.065
Skeletal muscle density	0.97 (0.91 to 1.03)	.313	0.97 (0.90 to 1.05)	.470
Skeletal muscle gauge	0.99 (0.93 to 1.06)	.798	1.02 (0.94 to 1.10)	.659
Subcutaneous adipose tissue index	0.96 (0.90 to 1.02)	.181	0.98 (0.92 to 1.05)	.662
Visceral adipose tissue index	1.06 (1.00 to 1.13)	.049	1.07 (1.00 to 1.14)	.051

Abbreviations: CI = confidence interval; HR = hazard ratio.

a Corrected for age, sex, serum lactate dehydrogenase, presence of brain metastases (absent vs asymptomatic vs symptomatic), and liver metastases, Eastern Cooperative Oncology group performance status and number of affected organs.

b Hazard ratios for skeletal muscle index, density, and gauge, and subcutaneous and visceral adipose tissue indices are provided per SD increase.

**Table 3.** Univariable and multivariable Cox proportional hazard models for overall survival.

	Univariable		Multivariable^a^	
	HR^b^ (95% CI)	*P*	HR^b^ (95% CI)	*P*
Body mass index				
Underweight	1.49 (0.85 to 2.59)	.162	1.56 (0.89 to 2.72)	.123
Normal	1.00		1.00	
Overweight	0.95 (0.80 to 1.12)	.523	1.00 (0.84 to 1.18)	.962
Obese	1.00 (0.81 to 1.23)	.977	1.17 (0.95 to 1.45)	.144
Skeletal muscle index	0.97 (0.90 to 1.05)	0.427	1.03 (0.94 to 1.13)	.526
Skeletal muscle density	0.85 (0.79 to 0.92)	<.001	0.88 (0.81 to 0.97)	.007
Skeletal muscle gauge	0.86 (0.80 to 0.93)	<.001	0.91 (0.82 to 0.998)	.047
Subcutaneous adipose tissue index	0.94 (0.87 to 1.01)	.095	1.02 (0.94 to 1.10)	.684
Visceral adipose tissue index	1.14 (1.06 to 1.22)	<.001	1.12 (1.04 to 1.22)	.004

Abbreviations: CI = confidence interval; HR = hazard ratio.

a Corrected for age, sex, serum lactate dehydrogenase, presence of brain metastases (absent vs asymptomatic vs symptomatic) and liver metastases, Eastern Cooperative Oncology group performance status and number of affected organs.

b Hazard ratios for skeletal muscle index, density, and gauge, and subcutaneous and visceral adipose tissue indices are provided per SD increase.

**Table 4.** Univariable and multivariable Cox proportional hazard models for melanoma-specific survival.

	Univariable		Multivariable^a^	
	HR^b^ (95% CI)	*P*	HR^b^ (95% CI)	*P*
Body mass index				
Underweight	1.35 (0.69 to 2.63)	.376	1.36 (0.69 to 2.69)	.372
Normal	1.00		1.00	
Overweight	0.92 (0.76 to 1.12)	.436	0.98 (0.80 to 1.19)	.842
Obese	0.91 (0.71 to 1.17)	.448	1.09 (0.85 to 1.41)	.502
Skeletal muscle index	1.01 (0.93 to 1.10)	.815	1.09 (0.98 to 1.21)	.122
Skeletal muscle density	0.90 (0.82 to 0.98)	.021	0.88 (0.80 to 0.98)	.018
Skeletal muscle gauge	0.92 (0.84 to 1.01)	.074	0.93 (0.83 to 1.04)	.191
Subcutaneous adipose tissue index	0.93 (0.85 to 1.02)	.134	1.02 (0.93 to 1.12)	.714
Visceral adipose tissue index	1.08 (0.99 to 1.17)	.094	1.10 (0.997 to 1.21)	.059

Abbreviations: CI = confidence interval; HR = hazard ratio.

a Corrected for age, sex, serum lactate dehydrogenase, presence of brain metastases (absent vs asymptomatic vs symptomatic) and liver metastases, Eastern Cooperative Oncology group performance status, and number of affected organs.

b Hazard ratios for skeletal muscle index, density, and gauge, and subcutaneous and visceral adipose tissue indices are provided per SD increase.

### CT-derived body composition metrics

All body composition metrics were significantly correlated with each other (data not shown). Of note is the negative correlation between skeletal muscle index and density (*r* = −0.14). Body composition differed significantly between male and female sex (Table S4). Significant associations with outcomes were observed for 3 of the 5 CT-derived body composition metrics. First, higher skeletal muscle density was associated with better OS (multivariable HR = 0.88 per SD increase, 95% CI = 0.81 to 0.97, Table 3) and MSS (multivariable HR = 0.88 per SD increase, 95% CI = 0.80 to 0.98, Table 4). Second, higher skeletal muscle gauge was associated with better OS (multivariable HR = 0.91 per SD increase, 95% CI = 0.82 to 0.998, Table 3). Third, higher visceral adipose tissue index was associated with worse OS (multivariable HR = 1.12 per SD increase, 95% CI = 1.04 to 1.22, Table 3), with similar but statistically nonsignificant trends for PFS (multivariable HR = 1.07 per SD increase, 95% CI = 1.00 to 1.14, Table 2) and MSS (multivariable HR = 1.10 per SD increase, 95% CI = 0.997 to 1.21, Table 4). No significant associations were observed between skeletal muscle index or subcutaneous adipose tissue index and survival outcomes.

Results were similar in subgroups of patients treated with anti-PD1 and combination therapy (Tables S5 and S6) and in subgroups of patients who underwent a noncontrast or contrast-enhanced CT scan (results not shown). When defining groups into having sarcopenia or myosteatosis, no associations were identified with OS (univariable HR = 1.12, 95% CI = 0.96 to 1.31 and univariable HR = 1.07, 95% CI = 0.90 to 1.27, respectively), PFS (data not shown), or MSS (data not shown).

## Discussion

The contributions of this work are 3-fold. First, we demonstrate that underweight patients experience significantly worse PFS. Second, we find no evidence supporting an association between obesity and improved survival outcomes. Third, we show that higher SMD and lower visceral adipose tissue index correlate with improved OS.

Progression-free survival was significantly worse in underweight patients. Surprisingly, this association remained significant when adjusted for liver and (symptomatic) brain metastasis, stage of disease, performance status, and level of LDH in multivariable analysis, suggesting that the association between underweight BMI and survival is independent of these patient and tumor characteristics. These result must be interpreted with care due to the small numbers (*n* = 21) in the underweight group. Also, residual bias cannot be fully ruled out.

We found no association between obesity and better treatment outcomes or survival, when measured as BMI, or as visceral or subcutaneous adipose tissue index. In contrast, we observed worse survival in patients with more visceral adipose tissue, the type of fat most associated with inflammation.^23^ These findings are in line with the meta-analysis by Roccuzzo et al.,^8^ which found no significant association between higher BMI and survival outcomes in melanoma. This meta-analysis thereby differs in its conclusion from earlier meta-analyses, a fact that can be explained by the inclusion of larger studies that were not yet published during these earlier analyses.

In the analyses of muscle compartments using CT-based body composition, we found that SMD was associated with improved OS. Skeletal muscle index, however, was not associated with improved survival, also not while using predefined cutoffs for sarcopenia. These results are in line with other studies investigating the association between SMD, SMI, and ICI outcomes in melanoma.^9^^,^^24^^,^^25^

There are multiple explanations for the association between SMD, VATI, and survival outcomes. One possibility is that SMD decreases due to cancer-related cachexia, a condition linked to factors such as low albumin levels and increased clearance of anti-PD1 antibodies.^26^ Both of these factors are associated with reduced treatment response and poorer survival.^27-29^ A second explanation is that higher SMD and lower VATI might be the result of increased physical activity, which is associated with prolonged survival in ICI-treated patients.^30^ In addition, visceral fat might lead to a dysregulation of the body’s immune system, leading to a diminished antitumor response and worse treatment effects.^31^^,^^32^ Visceral fat increase may also reflect dietary patterns, as suggested by the link between a Mediterranean diet and improved response to ICI therapy.^33^ However, conflicting literature on these topics exists, warranting the need for future research to confirm these associations and to determine the underlying causal mechanisms. A direct effect on survival due to lower antibody concentration is less likely, as body composition has minimal impact on antibody exposure and no clear dose-response relationship exists for anti-PD1 ICI therapy.^28^^,^^34^

This study contributes to previous evidence in 2 important ways. First, it adds the largest cohort collected on this topic to date and thereby strengthens the conclusion of the meta-analysis by Roccuzzo et al. regarding obesity.^8^ Second, it provides a more fine-grained view of body composition through the use of CT-derived body composition metrics. This is particularly relevant in the case of visceral adipose tissue, where our findings suggest a negative association with survival, rather than a positive one as was suggested by earlier findings on BMI.

A limitation is the exclusion of otherwise eligible patients due to unavailable data. Approximately 25% of eligible patients were excluded due to lack of required data. We consider the risk of selection bias is limited, as differences in patient characteristics between included and excluded patients were small. Furthermore, the correction of SMD for the presence of contrast is likely to be imperfect. This correction assumes that the mean and SD of the true skeletal muscle density is the same for patients who underwent contrast-enhanced and no-contrast baseline scans. This may not be the case, given the difference in patient characteristics between the 2 groups. Given the consistent results in the subgroup analyses, we think it is unlikely that this imperfect correction would have significantly influenced the results.

Considering the availability of (fluorodeoxyglucose positron emission tomography [FDG-PET]) CT scans for every patient with advanced melanoma, SMD and VATI could be used for prognostic survival estimation in a multimodal prediction model. However, currently multiple software tools and acquisition protocols are used, as well as multiple ways to define body composition metrics.^35^ To facilitate implementation, standardization of body metrics definition is needed.

In conclusion, underweight BMI, and more visceral adipose tissue and lower SMD as determined by CT-derived body composition are associated with worse survival outcomes in ICI-treated advanced melanoma patients. This association is independent of known predictive predictors such as LDH, ECOG performance status, and the presence of brain metastasis. No association was found between obesity and survival outcomes.

## Supplementary Material

djaf039_Supplementary_Data

## Data Availability

Data produced in the present work is not available due to confidentiality agreements.
