# Supplementary material for: Body composition and checkpoint inhibitor treatment outcomes in advanced melanoma: a multicenter cohort study
Source: J Natl Cancer Inst. 2025 Feb 20;117(6):1245–52. doi: 10.1093/jnci/djaf039 (PMC12145918; doi:10.1093/jnci/djaf039)

Supplementary Table 1 - Characteristics and outcomes of included versus excluded patients

		Included	Excluded
n		1471	470
Age, mean (SD)		65.1 (13.0)	65.0 (13.9)
Sex, n (%)	Female	579 (39.4)	167 (35.5)
	Male	892 (60.6)	303 (64.5)
Therapy, n (%)	Anti-PD1	942 (64.0)	300 (63.8)
	Ipilimumab & Nivolumab	529 (36.0)	170 (36.2)
Scan type, n (%)	Contrast-enhanced	860 (58.5)	53 (66.2)
	No contrast	611 (41.5)	27 (33.8)
Stage, n (%)	IIIC	131 (8.9)	33 (7.0)
	IV M1a	130 (8.8)	37 (7.9)
	IV M1b	217 (14.8)	77 (16.4)
	IV M1c	639 (43.4)	197 (41.9)
	IV M1d	344 (23.4)	119 (25.3)
	missing	10 (0.7)	7 (1.5)
ECOG performance status, n (%)	0	798 (54.2)	239 (50.9)
	1	489 (33.2)	177 (37.7)
	2-4	110 (7.5)	31 (6.6)
	missing	74 (5.0)	23 (4.9)
Brain metastases, n (%)	absent	952 (64.7)	308 (65.5)
	asymptomatic	212 (14.4)	66 (14.0)
	symptomatic	132 (9.0)	53 (11.3)
	missing	175 (11.9)	43 (9.1)
Liver metastases, n (%)	absent	939 (63.8)	314 (66.8)
	present	379 (25.8)	112 (23.8)
	missing	153 (10.4)	44 (9.4)
LDH, n (%)	normal	1013 (68.9)	302 (64.3)
	1-2x ULN	330 (22.4)	130 (27.7)
	>2x ULN	110 (7.5)	31 (6.6)
	missing	18 (1.2)	7 (1.5)
Number of affected organs, n (%)	<3	886 (60.2)	277 (58.9)
	>2	585 (39.8)	193 (41.1)
Body Mass Index, n (%)	underweight	21 (1.4)	0
	normal	604 (41.1)	30 (39.5)
	overweight	586 (39.8)	26 (34.2)
	obese	260 (17.7)	20 (26.3)
Skeletal Muscle Index, median [Q1,Q3]		45.8 [40.8,50.6]	44.4 [38.7,50.0]
Skeletal Muscle Density, median [Q1,Q3]		30.9 [24.2,37.8]	30.7 [23.1,37.1]
Skeletal Muscle Gauge, median [Q1,Q3]		1316.3 [990.0,1804.9]	1348.8 [970.1,1741.0]
Subcutaneous Adipose Tissue Index, median [Q1,Q3]		44.0 [29.9,66.4]	51.7 [37.6,72.9]
Visceral Adipose Tissue Index, median [Q1,Q3]		45.3 [22.7,80.1]	47.6 [26.6,73.3]
Median overall survival (months)		38.1	29.1
Median progression-free survival (months)		9.1	8.1
Median melanoma-specific survival (months)		Not reached	47.1

Supplementary Table 2 - Characteristics and outcomes of patients who received anti-PD1 versus combination therapy

		Anti-PD1	Ipilimumab & Nivolumab
n		942	529
Age, mean (SD)		67.0 (12.8)	61.6 (12.6)
Sex, n (%)	Female	380 (40.3)	199 (37.6)
	Male	562 (59.7)	330 (62.4)
Scan type, n (%)	Contrast-enhanced	538 (57.1)	322 (60.9)
	No contrast	404 (42.9)	207 (39.1)
Stage, n (%)	IIIC	113 (12.0)	18 (3.4)
	IV M1a	112 (11.9)	18 (3.4)
	IV M1b	184 (19.5)	33 (6.2)
	IV M1c	397 (42.1)	242 (45.7)
	IV M1d	130 (13.8)	214 (40.5)
	missing	6 (0.6)	4 (0.8)
ECOG performance status, n (%)	0	537 (57.0)	261 (49.3)
	1	291 (30.9)	198 (37.4)
	2-4	62 (6.6)	48 (9.1)
	missing	52 (5.5)	22 (4.2)
Brain metastases, n (%)	absent	672 (71.3)	280 (52.9)
	asymptomatic	80 (8.5)	132 (25.0)
	symptomatic	50 (5.3)	82 (15.5)
	missing	140 (14.9)	35 (6.6)
Liver metastases, n (%)	absent	638 (67.7)	301 (56.9)
	present	177 (18.8)	202 (38.2)
	missing	127 (13.5)	26 (4.9)
LDH, n (%)	normal	749 (79.5)	264 (49.9)
	1-2x ULN	157 (16.7)	173 (32.7)
	>2x ULN	25 (2.7)	85 (16.1)
	missing	11 (1.2)	7 (1.3)
Number of affected organs, n (%)	<3	652 (69.2)	234 (44.2)
	>2	290 (30.8)	295 (55.8)
Body Mass Index, n (%)	underweight	15 (1.6)	6 (1.1)
	normal	365 (38.7)	239 (45.2)
	overweight	380 (40.3)	206 (38.9)
	obese	182 (19.3)	78 (14.7)
Skeletal Muscle Index, median [Q1,Q3]		44.0 [38.7,50.3	45.1 [39.0,49.8]
Skeletal Muscle Density, median [Q1,Q3]		29.7 [22.4,36.1]	32.2 [24.7,38.7]
Skeletal Muscle Gauge, median [Q1,Q3]		1318.0 [933.6,1701.4]	1419.7 [1030.6,1804.8]
Subcutaneous Adipose Tissue Index, median [Q1,Q3]		52.7 [38.8,73.1]	48.9 [35.3,72.2]
Visceral Adipose Tissue Index, median [Q1,Q3]		52.0 [28.9,76.1]	42.1 [22.5,68.6]
Median overall survival (months)		34.2	37.7
Median progression-free survival (months)		9.9	6.5
Median melanoma-specific survival (months)		66.5	51.2

Supplementary Table 3 - Characteristics and outcomes of patients according to BMI subgroup

		underweight	normal	overweight	obese
n		21	604	586	260
Age, mean (SD)		61.6 (17.2)	65.2 (14.2)	65.3 (12.2)	64.5 (11.4)
Sex, n (%)	Female	17 (81.0)	267 (44.2)	188 (32.1)	107 (41.2)
	Male	4 (19.0)	337 (55.8)	398 (67.9)	153 (58.8)
Therapy, n (%)	Anti-PD1	15 (71.4)	365 (60.4)	380 (64.8)	182 (70.0)
	Ipilimumab & Nivolumab	6 (28.6)	239 (39.6)	206 (35.2)	78 (30.0)
Scan type, n (%)	Contrast-enhanced	15 (71.4)	351 (58.1)	336 (57.3)	158 (60.8)
	No contrast	6 (28.6)	253 (41.9)	250 (42.7)	102 (39.2)
Stage, n (%)	IIIC	2 (9.5)	57 (9.4)	39 (6.7)	33 (12.7)
	IV M1a	1 (4.8)	37 (6.1)	63 (10.8)	29 (11.2)
	IV M1b	2 (9.5)	78 (12.9)	88 (15.0)	49 (18.8)
	IV M1c	8 (38.1)	286 (47.4)	242 (41.3)	103 (39.6)
	IV M1d	7 (33.3)	146 (24.2)	148 (25.3)	43 (16.5)
	missing	1 (4.8)		6 (1.0)	3 (1.2)
ECOG performance status, n (%)	0	11 (52.4)	321 (53.1)	334 (57.0)	132 (50.8)
	1	6 (28.6)	208 (34.4)	175 (29.9)	100 (38.5)
	2-4	3 (14.3)	53 (8.8)	38 (6.5)	16 (6.2)
	missing	1 (4.8)	22 (3.6)	39 (6.7)	12 (4.6)
Brain metastases, n (%)	absent	12 (57.1)	388 (64.2)	375 (64.0)	177 (68.1)
	asymptomatic	6 (28.6)	84 (13.9)	96 (16.4)	26 (10.0)
	symptomatic	1 (4.8)	62 (10.3)	52 (8.9)	17 (6.5)
	missing	2 (9.5)	70 (11.6)	63 (10.8)	40 (15.4)
Liver metastases, n (%)	absent	9 (42.9)	368 (60.9)	384 (65.5)	178 (68.5)
	present	10 (47.6)	175 (29.0)	151 (25.8)	43 (16.5)
	missing	2 (9.5)	61 (10.1)	51 (8.7)	39 (15.0)
LDH, n (%)	normal	10 (47.6)	410 (67.9)	412 (70.3)	181 (69.6)
	1-2x ULN	8 (38.1)	138 (22.8)	123 (21.0)	61 (23.5)
	>2x ULN	3 (14.3)	51 (8.4)	42 (7.2)	14 (5.4)
	missing		5 (0.8)	9 (1.5)	4 (1.5)
Number of affected organs, n (%)	<3	12 (57.1)	349 (57.8)	355 (60.6)	170 (65.4)
	>2	9 (42.9)	255 (42.2)	231 (39.4)	90 (34.6)
Skeletal Muscle Index, median [Q1,Q3]		35.6 [32.9,36.8]	41.2 [36.7,46.6]	45.8 [40.9,51.6]	49.3 [42.7,55.6]
Skeletal Muscle Density, median [Q1,Q3]		39.0 [30.6,42.5]	33.0 [26.1,39.9]	30.2 [22.8,35.9]	25.7 [19.1,32.2]
Skeletal Muscle Gauge, median [Q1,Q3]		1349.9 [1058.2,1431.8]	1384.0 [1006.8,1763.4]	1372.4 [976.2,1749.9]	1253.2 [873.0,1651.5]
Subcutaneous Adipose Tissue Index, median [Q1,Q3]		19.6 [14.4,31.7]	40.3 [29.8,51.9]	56.5 [43.9,73.8]	88.4 [64.3,119.5]
Visceral Adipose Tissue Index, median [Q1,Q3]		8.4 [4.8,15.6]	28.3 [15.5,45.2]	56.8 [38.5,76.3]	83.3 [63.4,105.5]
Median overall survival (months)		11.6	38.2	41.4	32.9
Median progression-free survival (months)		3	8.7	9.8	7.7
Median melanoma-specific survival (months)		18.5	not reached	not reached	66.5

Supplementary Table 4 – Body composition metrics for male and female sex.

Metric	Sex	
	Male (median [Q1 - Q3])	Female (median [Q1 - Q3])
SMI	47.98 [43.95 - 52.85]	38.60 [35.29 - 42.52]
SMD	31.65 [24.99 - 37.91]	28.14 [21.37 - 35.56]
SMG	1553.14 [1140.85 - 1899.03]	1090.16 [808.30 - 1417.15]
SATI	45.50 [33.94 - 59.03]	69.59 [47.08 - 94.58]
VATI	58.91 [35.10 - 84.55]	33.45 [16.42 - 53.16]
BMI	25.93 [24.05 - 28.40]	25.10 [22.59 - 28.69]

Supplementary Table 5 - Univariate and multivariate Cox proportional hazards models for overall survival in subgroup of patients treated with anti-PD1 (N=942)

		**Univariate**		**Multivariate***	
		HR** (95% CI)	p-value	HR** (95% CI)	p-value
Body Mass Index (categorical)	underweight	1.19 (0.61 - 2.32)	0.610	1.18 (0.59 - 2.34)	0.645
	normal	1.00		1.00	
	overweight	0.93 (0.76 - 1.14)	0.495	0.98 (0.79 - 1.20)	0.819
	obese	0.93 (0.73 - 1.20)	0.592	1.14 (0.88 - 1.48)	0.310
Body Mass Index (continuous)		0.99 (0.97 - 1.01)	0.275	1.01 (0.99 - 1.03)	0.432
Skeletal Muscle Index		0.95 (0.87 - 1.04)	0.270	0.99 (0.89 - 1.11)	0.926
Skeletal Muscle Density		0.84 (0.76 - 0.92)	0.000	0.88 (0.79 - 0.98)	0.022
Skeletal Muscle Gauge		0.85 (0.77 - 0.93)	0.001	0.89 (0.79 - 1.01)	0.061
Subcutaneous Adipose Tissue Index		0.89 (0.81 - 0.98)	0.022	0.99 (0.88 - 1.10)	0.813
Visceral Adipose Tissue Index		1.13 (1.04 - 1.23)	0.006	1.08 (0.98 - 1.19)	0.119

*Corrected for age, sex, serum lactate dehydrogenase, presence of brain metastases (absent vs. asymptomatic vs. symptomatic) and liver metastases, Eastern Cooperative Oncology group performance status and number of affected organs. Abbreviations: HR=Hazard Rate Ratio, CI=Confidence Interval

**Hazard rate ratios for skeletal muscle index, density and gauge, and subcutaneous and visceral adipose tissue index are provided per standard deviation increase.

Supplementary Table 6 - Univariate and multivariate Cox proportional hazards models for overall survival in subgroup of patients treated with ipilimumab plus nivolumab (N=529)

		Univariate		Multivariate*	
		HR** (95% CI)	p-value	HR** (95% CI)	p-value
Body Mass Index (categorical)	underweight	3.19 (1.17 - 8.69)	0.024	2.23 (0.82 - 6.45)	0.115
	normal	1.00		1.00	
	overweight	0.97 (0.73 - 1.30)	0.84	0.97 (0.72 - 1.30)	0.822
	obese	1.14 (0.78 - 1.68)	0.499	1.19 (0.80 - 1.76)	0.391
Body Mass Index (continuous)		1.00 (0.97 - 1.03)	0.939	1.01 (0.98 - 1.04)	0.613
Skeletal Muscle Index		1.00 (0.88 - 1.15)	0.951	1.14 (0.95 - 1.36)	0.152
Skeletal Muscle Density		0.86 (0.75 - 0.98)	0.024	0.89 (0.76 - 1.04)	0.149
Skeletal Muscle Gauge		0.88 (0.77 - 1.01)	0.069	0.94 (0.80 - 1.12)	0.517
Subcutaneous Adipose Tissue Index		1.00 (0.89 - 1.13)	0.953	1.05 (0.93 - 1.19)	0.465
Visceral Adipose Tissue Index		1.18 (1.04 - 1.34)	0.012	1.23 (1.07 - 1.42)	0.005

*Corrected for age, sex, serum lactate dehydrogenase, presence of brain metastases (absent vs. asymptomatic vs. symptomatic) and liver metastases, Eastern Cooperative Oncology group performance status and number of affected organs. Abbreviations: HR=Hazard Rate Ratio, CI=Confidence Interval

**Hazard rate ratios for skeletal muscle index, density and gauge, and subcutaneous and visceral adipose tissue index are provided per standard deviation increase.

Supplementary Figure 1 – Flowchart of the inclusion process

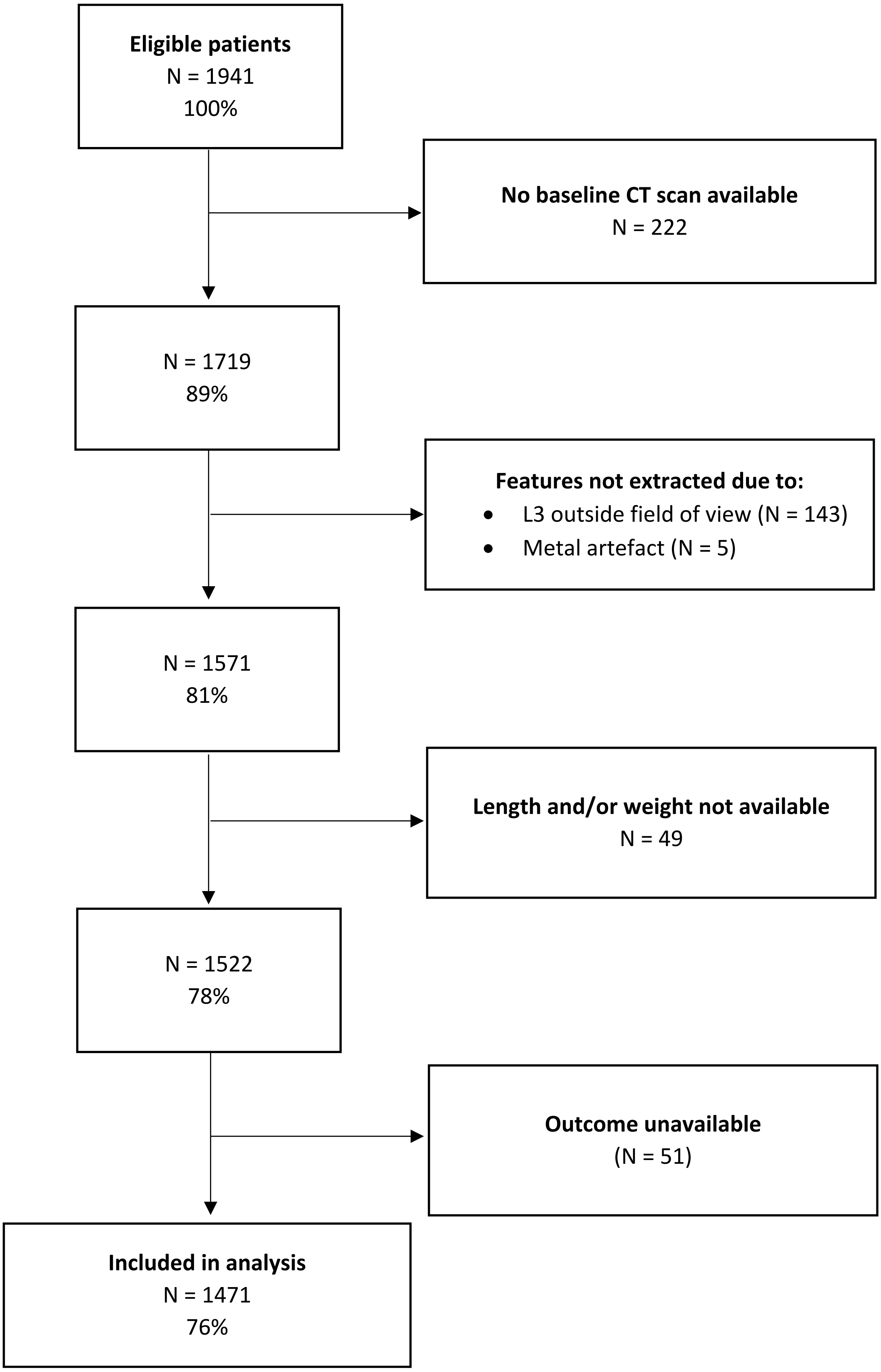

Supplement: djaf039_Supplementary_Data [file djaf039_supplementary_data.pdf]
